# Comparative Genomics of the Major Histocompatibility Complex (MHC) of Felids

**DOI:** 10.3389/fgene.2022.829891

**Published:** 2022-03-02

**Authors:** Martin Plasil, Jan Futas, April Jelinek, Pamela A. Burger, Petr Horin

**Affiliations:** ^1^ Research Group Animal Immunogenomics, Ceitec Vetuni, University of Veterinary Sciences Brno, Brno, Czech Republic; ^2^ Department of Animal Genetics, Faculty of Veterinary Medicine, University of Veterinary Sciences Brno, Brno, Czech Republic; ^3^ Research Institute of Wildlife Ecology, University of Veterinary Medicine Vienna, VIA, Vienna, Austria

**Keywords:** major histocompatibility complex, comparative genomics, felidae, domestic cat, *Felis catus*, genetic diversity, conservation genetics, natural killer cell receptor ligands

## Abstract

This review summarizes the current knowledge on the major histocompatibility complex (MHC) of the family Felidae. This family comprises an important domestic species, the cat, as well as a variety of free-living felids, including several endangered species. As such, the Felidae have the potential to be an informative model for studying different aspects of the biological functions of MHC genes, such as their role in disease mechanisms and adaptation to different environments, as well as the importance of genetic diversity for conservation issues in free-ranging or captive populations. Despite this potential, the current knowledge on the MHC in the family as a whole is fragmentary and based mostly on studies of the domestic cat and selected species of big cats. The overall structure of the domestic cat MHC is similar to other mammalian MHCs following the general scheme “centromere-MHC class I-MHC class III-MHC class II” with some differences in the gene contents. An unambiguously defined orthologue of the non-classical class I *HLA-E* gene has not been identified so far and the class II DQ and DP genes are missing or pseudogenized, respectively. A comparison with available genomes of other felids showed a generally high level of structural and sequence conservation of the MHC region. Very little and fragmentary information on *in vitro* and/or *in vivo* biological functions of felid MHC genes is available. So far, no association studies have indicated effects of MHC genetic diversity on a particular disease. No information is available on the role of MHC class I molecules in interactions with Natural Killer (NK) cell receptors or on the putative evolutionary interactions (co-evolution) of the underlying genes. A comparison of complex genomic regions encoding NK cell receptors (the Leukocyte Receptor Complex, LRC and the Natural Killer Cell Complex, NKC) in the available felid genomes showed a higher variability in the NKC compared to the LRC and the MHC regions. Studies of the genetic diversity of domestic cat populations and/or specific breeds have focused mainly on *DRB* genes. Not surprisingly, higher levels of MHC diversity were observed in stray cats compared to pure breeds, as evaluated by *DRB* sequencing as well as by MHC-linked microsatellite typing. Immunogenetic analysis in wild felids has only been performed on MHC class I and II loci in tigers, Namibian leopards and cheetahs. This information is important as part of current conservation tasks to assess the adaptive potential of endangered wild species at the human-wildlife interface, which will be essential for preserving biodiversity in a functional ecosystem.

## Felids as a Possible Model Mammalian Family for Studying the Biology of the Major Histocompatibility Complex

Genes located in the Major Histocompatibility Complex genomic region play important roles in multiple immune and non-immune processes of vertebrates (Murphy and Weaver, 2016). The current knowledge of the structure, functions, diversity and evolution of the mammalian MHC and its associations with important traits is mostly based on extensively studied model species, including humans and laboratory rodents. In addition, special attention has been paid to studying economically important domestic mammalian species, such as cattle, swine, horses, and sheep.^1^ On the other hand, less information is available on the MHC of other domestic animals, including dogs and cats, and our knowledge of the MHCs of wild and/or captive species is rather fragmentary.

Due to the critical importance of MHC molecules for survival, the evolution of the MHC reflects various selective pressures, especially those exerted by pathogens, which are considered to be one of the driving forces of evolution (Sironi et al., 2015). Comparative genomics represents an informative tool for studying the effects of selective pressures acting on MHC genes (Kelley et al., 2005a). Since different species live in different environments and are confronted with different pathogens, differences in the organization of the MHC region and especially in the MHC gene content can be observed not only between different classes of vertebrates, but also between families and even between species within a family. For this purpose, it is possible to select various informative models. Studies of entire families may provide better insight into evolutionary processes in a group of genetically related yet distinct species.

The family Felidae has the potential to be an informative model for such a purpose. It is a mammalian family with phylogenetic relationships defined based on standard zoological criteria as well as on molecular genetic and genomic data (Mattern and McLennan, 2000; Hassanin et al., 2021). It comprises a high number of species, including an important domestic species, the domestic cat (*Felis catus*), and a wide range of wild species living in different habitats. Many of these species exhibit different social structures and relationships, including mostly solitary species, species organized in packs, and captive populations in zoos. These differences imply differences in selective pressures due to variations in their environments, including pathogen richness, types of major pathogens present and opportunities for pathogen transmission and sharing. The domestic cat currently exists in hundreds of different pure breeds as well as in populations of free-living stray cats, supposed to be panmictic and genetically heterogenous around the world (Kurushima et al., 2013; Koch et al., 2016). Overlapping niches between different cat populations allow continual although limited horizontal gene flow (Kurushima et al., 2013). Several species, such as snow leopards, cheetahs, and tiger and leopard sub-species, are critically endangered due to their low population sizes and repeated bottlenecks.

Domestic, free-ranging, and captive Felidae are confronted with serious viral, bacterial and protozoan infections as well as with various types of ectoparasites (Chalkowski et al., 2019; Souza et al., 2021). Some of these pathogens, such as the retroviruses Feline Leukemia Virus (FLV) and Feline Immunodeficiency Virus (FIV) or the feline coronavirus (FCoV) are thoroughly studied experimental models and practically important agents causing fatal diseases (O’Brien et al., 2012). Despite the importance of infectious diseases for all felid species, their immune system has been studied only to a limited extent compared to other domestic species. Most of the information on immune functions that is available pertains to domestic cats.^2^ However, there are gaps in our knowledge of important immune cells and their functions even in this species. For example, feline NK cells have been only poorly characterized in terms of both their sub-population heterogeneity and their functions (Vermeulen et al., 2012). For the family Felidae as a whole, information on its immunogenome and the biological roles of immunity related (IR) genes, including the MHC, is mostly fragmentary and limited to isolated studies of rather specific problems in selected species.

The purpose of this review is to provide a survey of the current knowledge of different aspects of the MHC region in the family Felidae and to point out some gaps important from the perspective of future research and its possible applications.

## Comparative Major Histocompatibility Complex Genomics of Felids

The Major Histocompatibility Complex is considered to be one of the most complex regions of the mammalian genome (Kulski et al., 2002). This is especially true for the class I and class II subregions harboring clusters of genes encoding molecules involved in antigen presentation. The complexity of the MHC class I and II regions stems mainly from frequent structural rearrangements and duplications throughout the evolutionary history of MHC class I and II genes (Kumánovics et al., 2003; Yuhki et al., 2007). Due to this complexity, multiple methodological obstacles are encountered in studying the MHC genomic structure. Especially, the quality of MHC genomic maps and their annotation depends directly on the quality of available genomic resources, which in felids are mainly represented by genome assemblies at different stages of annotation. In this situation, the quality of genomes can be significantly improved by using long-read sequencing approaches (He et al., 2021).

**FIGURE 1:**
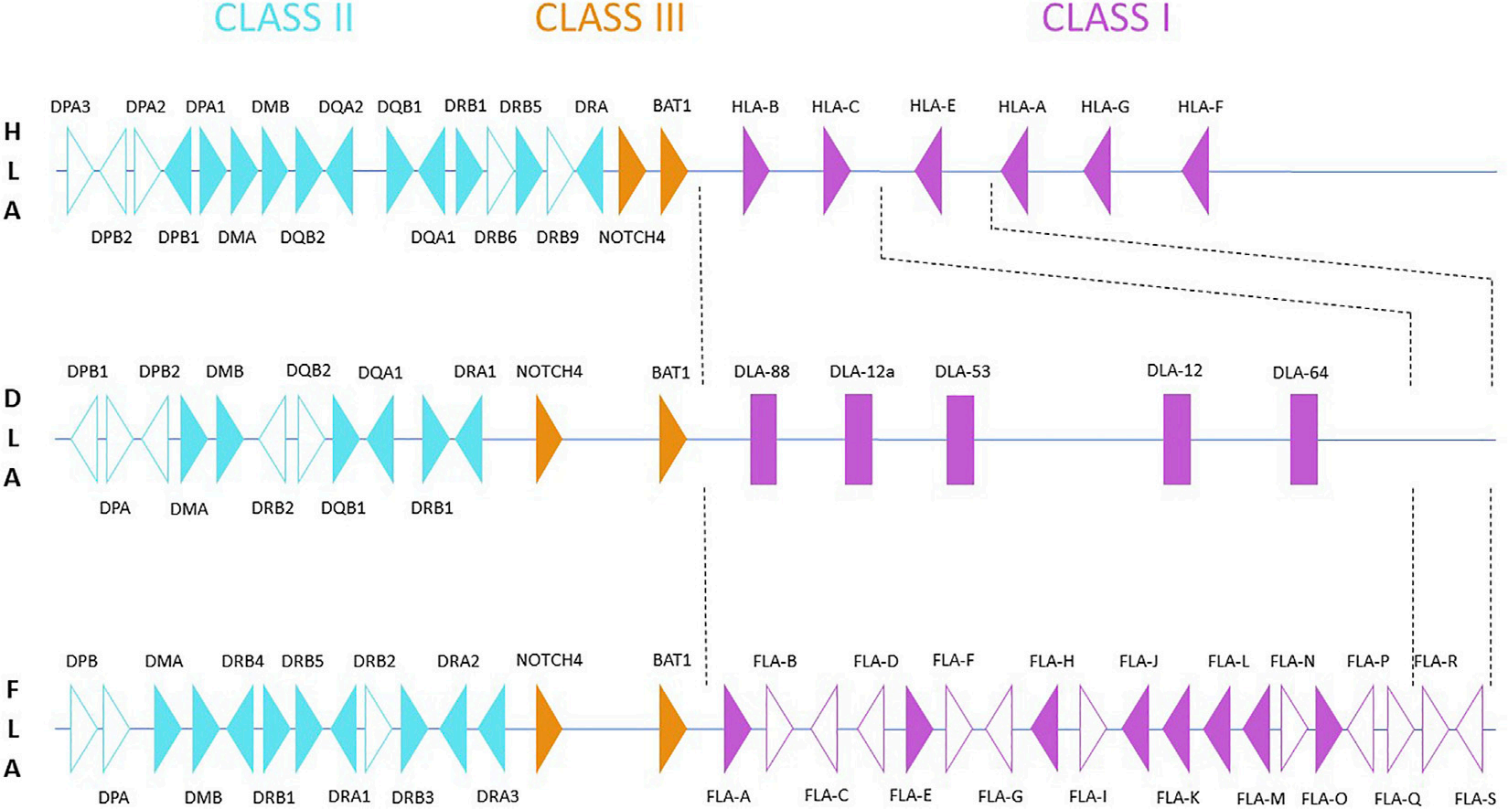
Schematic comparison of the *Felis catus* MHC region located on chromosome B2 (based on the sequence EU153401.1 by Yuhki et al., 2008) and compared with the human (HLA) and canine (DLA) MHC regions. Filled triangles represent potentially functional genes; unfilled triangles represent pseudogenes (based on Okano et al., 2020). DLA class I is presented as rectangles, since the original material does not provide gene orientation and original data is not available (Yuhki et al., 2007). The DLA class II map is based on Debenham et al., 2005, for HLA, it is based on the human genome GRCh38.p13.

Genomic chromosomal assemblies are currently available for domestic cat (*Felis catus*), jungle cat (*Felis chaus*), Geoffroy’s cat (*Leopardus geoffroyi*), Canada lynx (*Lynx canadensis*), lion (*Panthera leo*), tiger (*Panthera tigris*) and leopard cat (*Prionailurus bengalensis*) (Table 1). Besides the chromosome featuring assemblies, there are some felid species that were also sequenced, but are available only in the form of scaffolded assemblies (Table 2). Information about the genomic structure of the MHC region and its genes correlate with the amount of time available thus far for analyzing them in the respective genomic resources. The best picture of the MHC region in felids has been provided for the domestic cat, *Felis catus*. The general organization of its MHC region may serve as a potential model for other Felidae. It is comparable to the structure of other mammals (Figure 1). It follows the general scheme centromere-MHC class I-MHC class III-MHC class II (Okano et al., 2020). A closer comparison of currently available genomic data shows high levels of conservation of the MHC region between felids (Table 3). However, the gene content of the MHC subregions exhibits some significant differences from most other mammals.

**TABLE 1:** List of chromosomal level assemblies available to date (22-Nov-2021) for the family Felidae.

Common name	Scientific name	Assembly ID	Available since
Domestic cat	*Felis catus*	GCA_018350175.1	06-May-2021
		GCA_016509815.2	16-Dec-2020
		GCA_000181335.5	27-Apr-2006
		GCA_000003115.1	14-Jan-2009
Jungle cat	*Felis chaus*	GCA_019924945.1	14-Sep-2021
Geoffroy’s cat	*Leopardus geoffroyi*	GCA_018350155.1	13-May-2021
Canada lynx	*Lynx canadensis*	GCA_007474595.2	25-Jul-2019
Lion	*Panthera leo*	GCA_018350215.1	13-May-2021
		GCA_008795835.1	01-Oct-2019
Tiger	*Panthera tigris*	GCA_018350195.2	13-May-2021
Leopard cat	*Prionailurus bengalensis*	GCA_016509475.2	16-Dec-2020

**TABLE 2:** List of scaffold level assemblies available to date (22-Nov-2021) for the family Felidae.

Common name	Scientific name	Assembly ID	Available since
Cheetah	*Acinonyx jubatus*	GCA_003709585.1	22-Oct-2018
		GCA_001443585.1	13-Nov-2015
Caracal	*Caracal caracal*	GCA_016801355.1	02-Feb-2021
Black-footed cat	*Felis nigripes*	GCA_004023925.1	15-Jan-2019
Spanish lynx	*Lynx pardinus*	GCA_900661375.1	28-Mar-2019
Jaguar	*Panthera onca*	GCA_004023805.1	15-Jan-2019
Leopard	*Panthera pardus*	GCA_001857705.1	16-Nov-2016
Iriomote cat	*Prionailurus iriomotensis*	GCA_018403415.1	31-Mar-2021
Fishing cat	*Prionailurus viverrinus*	GCA_018119265.1	21-Apr-2021
Puma	*Puma concolor*	GCA_003327715.1	19-Jul-2018
		GCA_004123975.1	31-Jan-2019
Jaguarundi	*Puma yagouaroundi*	GCA_014898765.1	20-Oct-2020

**TABLE 3:** Similarities between the domestic cat MHC region (NC_058,372.1, range 28982338–32179391) and corresponding regions in genomes of other felids. The MHC region of each species was delimited based on its similarity with EU153401.1 (Yuhki et al., 2008). Nucleotide BLAST was used for retrieving similarity scores.

Species	GenBank ID	Similarity	Range	
*Felis chaus*	CM034423.1	99.20%	29150532	32323782
*Leopardus geoffroyi*	CM031398.1	98.05%	29080643	32415384
*Lynx canadensis*	CM017333.1	98.70%	28920729	32593795
*Panthera leo*	CM031454.1	97.80%	28859153	32050554
*Panthera tigris*	CM031435.1	98.01%	2,8941742	32161695
*Prionailurus bengalensis*	CM028183.1	97.67%	29050097	31806422

The MHC class I region contains at least nineteen different loci named *FLA-A* to *FLA-S*. Out of these, seven were confirmed as expressed across a range of tissue types (Holmes et al., 2013; Okano et al., 2020). As class I genes arise by a rapid birth-and-death process resulting in multiple new genes, an important diversification of MHC class I genes between mammalian families may occur. It seems that in the cat and the dog, the subregions corresponding to *HLA-A* and *HLA- E* in humans have been lost (Yuhki et al., 2007). Currently, *FLA-E*, *FLA-H* and *FLA-K* are classified as classical, while *FLA-A*, *FLA-J*, *FLA-L*, and *FLA-O* are considered non-classical class I genes (Holmes et al., 2021). Due to gene conversion and duplication events occurring during the process of diversification of class I genes, it is not possible to directly extrapolate from orthologous HLA or H-2 class I genes to define the classical/non-classical status of the FLA class I genes. This study highlighted some intrinsic difficulties related to this task. Especially, it was impossible to assign some of the retrieved sequences to specific “classical” loci. In this situation, it is also difficult to decipher whether and/or how the phylogenetically old and important functions of the HLA-E/H-2-Qa molecules have been substituted in cats (Grant et al., 2020). For the same reason, it is difficult to assess whether *HLA-E* genes are really missing in other felid genomes, although they have not been annotated there.

In contrast to dogs and humans (Figure 1), a complete absence of DQ genes and the presence of non-functional DP loci was observed within the MHC class II region of felids (Beck et al., 2001; Okano et al., 2020). It is difficult to assess whether differences observed in the presence of some functional class II genes in genomes of closely related families (e.g., Hyenidae or Herpestidae) are due to their different MHC class II evolution or whether they are artifacts resulting from the current quality of their genome assemblies and annotations. There is no information about the functional significance of these rather dramatic differences. It has been hypothesized that this loss of functionally important loci may be at least partly compensated by expansion of other class II loci, namely of DR genes (Yuhki et al., 2003). In the domestic cat, the MHC-DR sub-region consists of three *DRA* genes, four *DRB* genes and one *DRB* pseudogene (Yuhki et al., 2008; Okano et al., 2020). In agreement with the high degree of conservation of the MHC region observed amongst felids, DQ genes have not been annotated, while the status of DP sequences present in their genomes remains to be definitively determined.

Analysis of the genomes of other felids provided only fragmentary and descriptive information. There is currently no map of the MHC region available for other species of the family Felidae. The most information has been collected on the MHC class II (*DRB*) genes and their diversity in selected populations using either SNP or microsatellites markers. Such studies were performed for the leopard cat (*Prionailurus bengalensis*) (Saka et al., 2018), the Lynx genus (*Lynx lynx*, *Lynx pardinus*, *Lynx canadensis* and *Lynx rufus*) (Marmesat et al., 2017), the cheetah (*Acinonyx jubatus*) (Drake et al., 2004; Prost et al., 2020) and others (e.g., Wang et al., 2008; Wei et al., 2010). Since these data were produced mainly for characterizing the genetic diversity of selected free-ranging species, they are presented and discussed in the section dedicated to genetic diversity of wild felids.

For the nomenclature of MHC loci, it has been recommended to respect the rules set by the IPD-MHC database.^3^ Although there is currently no section dedicated to cats, the Comparative Major Histocompatibility Complex (MHC) Nomenclature Committee seeks to provide a guiding framework for allelic nomenclature across all non-human species.^4^

## *In vivo* and *in vitro* Functional Analyses of Major Histocompatibility Complex Genes in Felids

Functional capacities of feline MHC genes and molecules have so far been only poorly characterized both *in vitro* and *in vivo*. Rather specific *in vitro* models of selected diseases were studied, and no relevant *in vivo* studies have been performed. A naturally occurring model for lentivirus infection, the Feline immunodeficiency virus (FIV), was studied in the context of MHC functions. FIV-specific, MHC-restricted cytolytic T lymphocytes were identified in experimentally FIV-infected cats (Song et al., 1995). The mechanism of FIV-derived antigen presentation by an MHC class I molecule, FLA-E*01801, was characterized by Liang et al. (2018). Based on structural and biochemical experiments, the authors determined the crystal structure of FLA-E*01801, identified its binding motif and 125 FLA-E*01801-restricted nonapeptides derived from FIV. In several other models, e.g., Feline Infectious Peritonitis Virus (FIPV) infection (Watanabe et al., 2018), feline histiocytosis (Coste et al., 2019), and Cytauxzoonosis (Frontera-Acevedo and Sakamoto, 2015), the expression of MHC class genes was investigated. Altered expression of especially MHC class II genes was observed, but no specific features pertaining to the feline host were observed in these situations.

In contrast to most other domestic animal species, no studies focused on MHC and disease associations have been reported for cats. In our candidate gene study of feline coronavirus (FCoV) shedding, we observed an association of *FLA-DRB* polymorphisms with shedding patterns (Bubenikova et al., 2020). A few published GWAS studies did not identify significant hits within the MHC region, probably due to a focus on practically important traits and/or single-gene inherited disorders with no potential involvement of MHC genes (e.g., Gandolfi et al., 2012; Samaha et al., 2020).

Taken together, there is currently no comprehensive concept of the role of the feline MHC in various biological processes, which would allow the use of applications known from other species, such as the development of diagnostic and/or predictive markers for veterinary medicine and selective breeding as well as for conservation genetics. Based on the genetic structure of the MHC region and its genes, we may assume that in general it is similar to other mammalian species. However, comparative studies aim to mine important information from even subtle interspecific differences and peculiarities. Some examples, such as differences between cheetahs and leopards in the use of mechanisms of innate and adaptive immunity, suggesting different evolutionary pressures (Heinrich, 2018) show that comparisons of felids with other families as well as comparisons within the family still may be informative.

## A Reverse View Perspective on the Biology of Major Histocompatibility Complex: MHC Molecules as Ligands for NK Cell Receptors

### General Principles

In addition to their function in antigen presentation, MHC class I molecules have an important role in innate immunity as ligands for Natural Killer cell receptors (NKRs). NK cells are a key component of the innate immune system involved primarily but not exclusively in the elimination of intracellular pathogens, such as viruses and mycobacteria, as well as tumor cells (Caligiuri, 2008; Vivier et al., 2008; Freud et al., 2017). They are also important in the process of normal placental development both in humans and mice (Huhn et al., 2021). In order to perform all these functions, NK cells possess a variety of activating and inhibitory receptors; signals from these receptors are integrated to determine whether the NK cell is activated against a particular target. In many cases the ligands of the activating NKRs (aNKRs) have not yet been identified, whereas the ligands of the inhibitory NKRs (iNKRs) identified to date are most often MHC class I molecules (Lanier, 2005; Parham et al., 2012). In humans, 15 NK receptors recognizing HLA class I-specific ligands have been identified. Out of them, seven are activating and seven are inhibitory, and one receptor is capable of delivering both activating and inhibitory signals (Quatrini et al., 2021). These MHC/NKR interactions are important in situations where the expression of MHC molecules on the cell surface is altered or absent. In these cases, iNKRs do not bind ligand and therefore their inhibitory functions are not maintained, increasing the potential for NK cell activation and cytotoxicity.

There are three mechanisms by which the potential for NK cell activation may be increased in the absence of iNKR/MHC-I binding. In “non-self” detection, NK cells target foreign cells that do not express host MHC class I molecules. “Missing self” detection occurs when MHC class I expression is downregulated on the host’s own cells, often as a strategy of immune evasion by virally infected or tumor cells. Viruses such as Epstein-Barr Virus (EBV), Cytomegalovirus (CMV), and human immunodeficiency virus (HIV) are known to decrease the expression of MHC class I loci in order to escape T cell responses. The third mechanism, “altered self” detection, primarily relies on the ability of aNKRs to bind stress-induced or tumor-associated proteins expressed by damaged or unhealthy host cells. However, this process may also involve the sensitivity of some iNKR/MHC class I interactions to the peptide bound by the MHC class I molecule (Carrillo-Bustamante et al., 2016). Thus, the role of MHC class I as ligand for iNKRs is crucial for the immune function of NK cells.

The distinction between classical and non-classical MHC class I genes and molecules is particularly important in the context of NKR-MHC interactions. Although called “non-classical” due to the later discovery of their function in immune mechanisms, human HLA-E and mouse Qa-1b class I molecules represent phylogenetically older and conserved mechanisms of self/non-self discrimination relative to “classical” MHC class I molecules. As such, they are less polymorphic, exhibit tissue-restricted expression, and bind a less diverse ligandome than classical class I molecules. In these species, they function as ligands of invariant NK cell receptors CD94/NKG2A to provide protection against “missing self” *via* “gross” MHC class I detection (Pietra et al., 2003).

MHC class I/iNKR interactions are important for the development of polymorphism of both MHC class I molecules and NKRs. In the context of the evolutionary arms race between pathogens and the immune system, pathogens may develop immune-escape strategies such as selective MHC class I downregulation and the expression of MHC decoy molecules. Such strategies create an evolutionary need for highly polymorphic “classical” HLA/H-2 molecules and their recognition by NK cells, which has resulted in the co-evolution of the MHC class I and NKR genes and eventually led to the presence of three complex genomic regions in mammals: the MHC, the LRC, and the NKC. Each of these regions is located on a different chromosome. Further, several types of structurally different NKRs recognizing the same classical MHC class I ligands have evolved. In multiple mammalian species, including humans, the killer cell immunoglobulin-like receptor (KIR) genes have expanded to cope with the variability of MHC class I molecules (Kelley et al., 2005b; Parham et al., 2010; Guethlein et al., 2015). These genes are contained in the LRC and encode molecules containing immunoglobulin-like domains. In rodents and equids, the lectin-like *Ly49* (*KLRA*) genes have expanded in the NKC (Wilhelm et al., 2002; Wilhelm and Mager, 2004; Futas and Horin, 2013; Rahim and Makrigiannis, 2015). In prosimians, expanded families of *CD94*/*NKG2A* (*KLRD*/*KLRC*) genes fulfill this function (Averdam et al., 2009). Moreover, in humans the co-evolution of MHC and KIR genes was demonstrated based on the non-random population distribution of MHC-KIR haplotype combinations assuming they have different adaptive values (Single et al., 2007). In agreement with these findings, associations between MHC-KIR haplotype combinations are associated with various human diseases (Khakoo and Carrington, 2006). The influence of MHC haplotypes on NKR diversity in cattle has also been demonstrated (Allan et al., 2015).

As pointed out in the previous section, the subregions corresponding to human *HLA-A* and *HLA-E* have been lost in the cat (Yuhki et al., 2007). Due to the complexity of the rapid birth-and-death process resulting in multiple new MHC class I genes, it is not possible to directly extrapolate from orthologous HLA or H-2 class I genes to define the classical/non-classical status of the FLA class I genes and thus to identify putative NKR ligands. Considering the strong structural conservation of class I molecules reflecting common functional demands, genetic features related to their function may be used to distinguish between classical and non-classical genes and molecules (Holmes et al., 2021). For this purpose, knowledge of their receptors can provide important information, and therefore not only the MHC but also NKR loci should be properly annotated in the genomes of felids and characterized.

### Current knowledge on Major Histocompatibility Complex molecules binding Natural Killer Cell Receptors in felids: the felid Natural Killer Cells

**FIGURE 2:**
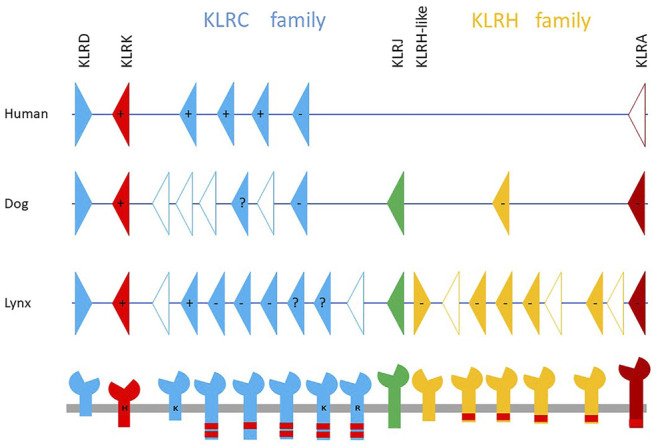
Organization of Natural Killer Complex genomic region in Canada lynx (*Lynx canadensis*) on chromosome B4 in comparison with the human and canine NKCs. Presumably functional genes (solid color triangles) along with pseudogenes/gene fragments (open triangles) are grouped in two families, KLRC (blue) and KLRH (yellow). The lower panel shows possible protein products, which may form functional cell receptors by homo- or heterodimerization. Their predicted signaling in terms of activation (+) or inhibition (−) is based on the presence of positively charged amino acid (H—histidine, K—lysine, R—arginine) in the transmembrane region or the immunoreceptor tyrosine-based inhibitory motifs (red rectangles) in cytoplasmic tail, respectively. When both features are present, the resulting signal is uncertain. Based on similarities to other mammalian species, all these putative proteins, except the products of the *KLRK* and *KLRJ* genes, could bind MHC class I ligands. The *KLRK* product can bind the stress-induced-self ligands MIC and ULBP; the ligands of KLRJ remain unknown. The organization of the canine Natural Killer Complex is based on the genome GCA_014441545.1, while the human NKC organization is based on Schwartz et al. (2017).

Unlike for other domestic animal species, such as ruminants (Schwartz et al., 2017), swine (Schwartz and Hammond, 2018), horses (Futas and Horin, 2013), and camels (Futas et al., 2019), so far no genes in felids have been defined as candidates potentially encoding NKR binding MHC molecules. Due to a lack of functional studies of NK cells, even the characterization of these receptors at the protein level is fragmentary. Based on comparative approaches, one can assume that receptors of this kind can be identified as expanded gene families in the NKC and/or the LRC. In felid genomes, the organization of the LRC on chromosome E2 appears conservative: there is only one *KIR* gene along with seven leukocyte immunoglobulin-like receptor (LILR) genes. On the other hand, the NKC seems rather variable in felid genomes, but this may be mainly due to different sequencing technologies and the quality of the felid genome assemblies. Only one *KLRA* gene accompanied by framing genes (*KLRD*, *KLRK*, *KLRJ*, *KLRH*-*like*) is shared by all published genomes. Two families of genes, *KLRC* and *KLRH*, have a variable number of members in felids. An example of the NKC on chromosome B4 in the Canada lynx compared with the human and canine NKC regions is shown in Figure 2. The lynx possesses eight *KLRC* genes and eight *KLRH* genes/gene fragments. The genomes of the domestic cat, tiger and lion, which are of comparable quality, contain seven, eight and fifteen *KLRC* genes respectively. The *KLRH* family has three genes in the cat and four genes in both the tiger and lion genomes. The expression status of each gene within these families, as well as that of single genes, is currently unknown.

## Polymorphism and Population Diversity of Major Histocompatibility Complex Genes in Felids

### Polymorphism of Major Histocompatibility Complex Genes in Domestic Cats

The extent of polymorphism of exon 2 (or exon2/3) and full-length gene sequences (Kennedy et al., 2002; Okano et al., 2020) as well as of MHC-linked microsatellite markers (Morris et al., 2014) was studied in domestic cats. It is only the advent of new sequencing techniques that allowed insight into the diversity of the feline MHC. A next generation sequencing (NGS)-based genotyping method revealed 32 FLA class I and 16 *FLA-DRB* sequences in two families of 20 domestic cats. Eight FLA Class I – *DRB* haplotypes were identified by a pedigree analysis of two cat families with five to eight FLA class I and two to three *FLA-DRB* transcribed loci per haplotype (Okano et al., 2020).

The haplotypes were generated by gene duplications, deletions, and rearrangements by genetic recombination (Okano et al., 2020). The major limitation of the findings pertaining to the polymorphism and diversity of the MHC in domestic cats is a sampling bias due to differences among local stray cat populations as well as between pure breeds. It thus seems that a large part of the existing variability has not been characterized yet.

Since the DQ sub-region is missing, and the *DRA* locus tends to exhibit only limited coding sequence polymorphism, at least in other species (Matern et al., 2020), multiple studies of MHC class II focused on exon 2 *DRB* sequences. In outbred domestic cats, *DRB* exon 2 exhibits a similar extent of polymorphism as in other species (Yuhki and O’Brien, 1997; Kennedy et al., 2002). Sixteen *FLA-DRB* nucleotide sequences were identified in cats by NGS in the above-mentioned study (Okano et al., 2020). Again, due to important differences between local populations of stray cats and to differences in the extent of inbreeding and genetic relatedness among particular samples of purebred cats, a direct comparison between different studies is usually not too informative. As expected in general terms, non-bred and stray cats exhibit higher levels of MHC diversity than purebred cats. Significant differences in the population diversity of MHC-linked microsatellite markers were observed between groups of outbred cats compared to Burmese cat populations in Australia, where the Burmese cats showed significantly lower diversity in the markers studied than their outbred counterparts (Morris et al., 2014). The extent of MHC polymorphism of other felid species could in most cases be evaluated based on studies aiming to map the genetic diversity of their free-ranging and/or captive populations.

### Genetic Diversity of Major Histocompatibility Complex in Wild Felids

Immune genetic analysis in wild felids has only been performed on specific genes of the adaptive immunity system, mainly MHC class I and II loci in tigers (Hendrickson et al., 2000; Pokorny et al., 2010), Namibian leopards and cheetahs (Castro-Prieto et al., 2011a, 2011b; Heinrich et al., 2017; Schwensow et al., 2019). In addition, immunological studies have investigated the constitutive innate immunity in Namibian cheetahs and leopards (Heinrich et al., 2017) and five other wild endangered felids (Heinrich et al., 2016). The results suggest that the immunocompetence of threatened felids such as the cheetah has been underestimated and its assessment ought to consider both innate and adaptive components of the immune system.

Tiger. The alpha-1 and alpha-2 domain of MHC class I and beta-1 domain of MHC class II *DRB* genes in 16 tiger specimens of different geographic origin were cloned and sequenced. While high variability in peptide-binding sites was detected, presumably resulting from positive selection, tigers in general exhibit a low number of MHC *DRB* alleles, similar to other endangered big cats (Pokorny et al., 2010). Significant geographic genetic population structure was found at the MHC class I loci among captive and wild tigers and phylogenetic analysis placed Sumatran tigers basal on the phylogenetic tree (Hendrickson et al., 2000).

Leopard. A study of genetic variation at the adaptively most important region of MHC class I and MHC class II-DRB genes in 25 free-ranging African leopards from Namibia investigated the mechanisms that generate and maintain MHC polymorphism in the species. The amino acid sequence variation in both MHC classes was higher than or similar to that of other felids, and there were signatures of positive selection that shaped the diversity of MHC class I and MHC class II-DRB loci during the evolutionary history of the species. A comparison of MHC class I and MHC class II-DRB sequences of the leopard to those of other felids revealed a trans-species mode of evolution (Castro-Prieto et al., 2011a). Comparing the functional competence of the humoral immune system in sympatric leopard and cheetah populations in Namibia revealed that cheetahs have a higher constitutive innate but lower induced innate and adaptive immunity than leopards (Heinrich et al., 2017). MHC class I and MHC class II-DRB diversity among wild and captive Indian leopards showed higher nucleotide diversity and a higher non-synonymous substitution rate in the wild group (Parmar et al., 2017).

Cheetah. Cheetahs are currently classified into four subspecies (*Acinonyx jubatus jubatus, A. j. soemmeringii, A. j. hecki* and *A. j. venaticus*) based on genetic data and geographical range (Krausman and Morales, 2005; Charruau et al., 2011; Kitchener et al., 2017). Past studies have found high disease susceptibility and depleted MHC diversity in cheetahs compared to other mammals, but they focused on small sample sets (O’Brien and Yuhki, 1999; Drake et al., 2004; Dobrynin et al., 2015), on captive individuals (Heeney et al., 1990), or on a single subspecies (Castro-Prieto et al., 2011b). Free-ranging cheetahs from Eastern and Southern Africa revealed strong disease resistance (Munson et al., 2004; Thalwitzer et al., 2010) even with low MHC diversity. It was shown that Namibian cheetahs have a stronger constitutive innate immunity than leopards (Heinrich et al., 2017), and harbor divergent MHC alleles with functionally distinct MHC supertypes across loci (Schwensow et al., 2019). In the latter article, the authors argue that in contrast to theoretical expectations, free-ranging cheetahs in Namibia show no signs of impaired immunocompetence or health. Their results support the hypothesis that species with a low MHC allelic diversity might be able to retain functional diversity not within but across loci, with the allelic composition influencing levels of MHC class I and class II gene expression. Despite low diversity, Namibian cheetahs seem to have an adequate response to current pathogen exposure, but it remains to be established whether this will suffice for future challenges of their immune system (Schwensow et al., 2019).

Snow leopard. The snow leopard (*Uncia uncia*) habitat ranges across mountainous areas of 12 countries in Central Asia (McCarthy and Chapron, 2003). The remaining populations are sparsely fragmented across these countries, with most residing in China and Mongolia. With a census number of barely 6,000 individuals and an effective population size of 2,500, the snow leopard is at risk of going through a massive genetic bottleneck and of losing even more diversity due to genetic drift, which acts faster on small populations. A recent phylogeographic study suggests the presence of three snow leopard subspecies across Asia (Janecka et al., 2017), isolating the Mongolian population as genetically distinct. In the light of these findings, it is expected that low allelic diversity will be found in Mongolian snow leopards. Immunogenetic studies have been restricted to three individuals (Wei et al., 2010).

Lynx. The MHC I and MHC II-DRB allelic repertoires in four lynx species, namely the bobcat (*Lynx rufus*), the Canada lynx (*L. canadensis*), the Eurasian lynx (*L. lynx*) and the Iberian lynx (*L. pardinus*) were characterized by many alleles shared among the species within the genus. The number of alleles found within individuals ranged between ten to sixteen (MHC I) and three to six (MHC II-DRB), with the lowest diversity found in the highly endangered Iberian lynx (Marmesat et al., 2017).

### Implications for Conservation Management in Free-Ranging and Captive Wild Felids

Pathogens pose a key threat to mammalian wildlife populations; according to the IUCN red list, 25% of carnivores are threatened and 8% of the 36 species in the family Felidae are threatened by diseases (Smith et al., 2009). Knowing the immune competence of threatened species is particularly important for conservation management because it is a critical aspect of disease resistance and the resilience of a population (Heinrich et al., 2016). Maintaining the diversity of immune response genes as functionally important genomic regions for local adaptation to pathogens contributes to the *in-situ* conservation of endangered populations and potentially to the survival of the species. Selective effects in an evolutionary arms race between host and pathogen appear to be the major driving forces for preserving MHC variation. As such, MHC variability reflects the evolutionary and adaptive potential of a population. Conservation genetic monitoring thus needs to assess the immunogenetic status in free-ranging or captive wild felid populations experiencing different environmental and pathogenic challenges (Sommer, 2005). Considering the ever-increasing impact of humans and their livestock on protected habitats, the adaptive potential of wild endangered species at the human-wildlife interface will be essential for preserving biodiversity in a functional ecosystem.

## Concluding Remarks

The family Felidae has the potential to be an informative model for studying different aspects of the biological functions of MHC genes. Despite this potential, the current knowledge of the MHC in the family as a whole is fragmentary and based mostly on studies of the domestic cat and selected species of big cats.

The overall structure of the domestic cat MHC is similar to other mammalian MHCs with some differences in the gene contents. An unambiguously defined orthologue of the non-classical class I *HLA-E* gene has not yet been identified and the class II DQ and DP genes are missing or pseudogenized, respectively.

A comparison with available genomes of other felids showed a generally high level of structural and sequence conservation of the MHC region as well as of the extent of MHC gene polymorphism.

Very little and fragmentary information on *in vitro* and/or *in vivo* biological functions of felid MHC genes is available. So far, no association studies have indicated effects of MHC genetic diversity on a particular disease. No information is available on the role of MHC class I molecules in interactions with Natural Killer (NK) cell receptors or on the putative evolutionary interactions (co-evolution) of the underlying genes.

Studies of the genetic diversity of domestic cat populations and/or breeds has focused mainly on *DRB* genes. Due to important differences between local populations of stray cats and differences in the extent of inbreeding and genetic relatedness among particular samples of purebred cats, a direct comparison between different studies is usually poorly informative. Not surprisingly, higher levels of MHC diversity have been observed in stray cats compared to pure breeds.

Immunogenetic analysis in wild felids has only been performed on MHC class I and II loci in tigers, Namibian leopards and cheetahs. This information is important as a part of current conservation tasks to assess the adaptive potential of wild endangered species at the human-wildlife interface, which will be essential for preserving biodiversity in a functional ecosystem.

## Footnotes

1
https://www.ebi.ac.uk/ipd/mhc/

2
https://www.uniprot.org/uniprot/?query=immunity+taxonomy%3Afelidae&sort=score#orgViewBy

3
https://www.ebi.ac.uk/ipd/mhc/

4
https://www.ebi.ac.uk/ipd/mhc/committee
